# Dusquetide: Reduction in oral mucositis associated with enduring ancillary benefits in tumor resolution and decreased mortality in head and neck cancer patients

**DOI:** 10.1016/j.btre.2017.05.002

**Published:** 2017-05-17

**Authors:** Mahesh Kudrimoti, Amarinthia Curtis, Samar Azawi, Francis Worden, Sanford Katz, Douglas Adkins, Marcelo Bonomi, Zack Scott, Jenna Elder, Stephen T. Sonis, Richard Straube, Oreola Donini

**Affiliations:** a Radiation Oncology, University of Kentucky, 800 Rose Street, Lexington, KY, 40536, USA; b Gibbs Cancer Center, Spartanburg Regional Hospital, 101 E Wood, Spartanburg, SC, 29303, USA; c Veteran’s Affairs Long Beach Hospital, 5901 E 7th Street, Mail Code 114A, Long Beach, CA, 98022, USA; d Department of Medicine, University of Michigan Health System,1500 E Medical Center Drive, Ann Arbor, MI, 48109, USA; e Department of Radiation Oncology, Willis-Knighton Cancer Center,2600 Kings Highway, Shreveport, LA, 71103, USA; f Division of Hematology and Oncology, Washington University, 660 South Euclid Avenue, Saint Louis, MO, 63110, USA; g Department of Hematology and Oncology, Wake Forest Health Sciences Medical Center, 1 Medical Center Blvd., Winston-Salem, NC, 27157, USA; h PharPoint Research, 5003 S Miami Blvd #100, Durham, NC, 27703, USA; i Oral Medicine and Diagnostic Services, Dana Farber/Harvard Cancer Center, Boston, MA, USA; j Biomodels LLC,313 Pleasant Street, Watertown, MA 02472, USA; k Soligenix Inc., 29 Emmons Drive, Suite C-10, Princeton, NJ, 08540, USA

**Keywords:** CRT, chemoradiation therapy, HNC, head and neck cancer, IDR, innate defense regulator, OM, oral mucositis, SOM, severe oral mucositis, dusquetide (PubChem CID: 71722017), Innate, Immune, Oral mucositis, Head and neck cancer, Cancer supportive care, Dusquetide

## Abstract

Innate immunity is a key component in the pathogenesis of oral mucositis, a universal toxicity of chemoradiation therapy (CRT). Dusquetide, a novel Innate Defense Regulator, has demonstrated both nonclinical and clinical efficacy in ameliorating severe oral mucositis (SOM). Long term follow-up studies from the Phase 2 clinical study evaluating dusquetide as a treatment for SOM in head and neck cancer (HNC) patients receiving CRT have now been completed. Extended analysis indicates that dusquetide therapy was well-tolerated and did not contribute to increased infection, tumor growth or mortality. Potential ancillary benefits of duquetide therapy were also identified.

## Highlights

• Dusquetide was well-tolerated during treatment and throughout the 12-month follow-up.
• Dusquetide did not interfere with tumor treatment.
• Dusquetide treated groups had less mortality that placebo treated groups through the 12-month follow-up.
• Dusquetide treated groups had fewer “non-fungal” infections than placebo treated groups.

## 1 Introduction

Interim results from a Phase 2 study evaluating a dose of 1.5 mg/kg of dusquetide as a treatment for severe oral mucositis (SOM) in head and neck cancer (HNC) patients receiving chemoradiation therapy (CRT) demonstrated a 50% decrease in the median duration of severe oral mucositis (SOM) in patients receiving at least 55 Gy irradiation [1]. Patients at higher risk for SOM showed even greater improvements (67%) relative to placebo, particularly in the treatment group receiving the 1.5 mg/kg dose of dusquetide [1]. Over this same treatment period, an increased number of patient classified as having a “complete tumor response” using the RECIST 1.1 tumor status system and a decreased “non-fungal” (i.e. bacterial) infection rate were also observed. Long term follow-up visits were conducted on these same patients for 12 months after the completion of CRT, with the last visits occurring in the fall of 2016.

There are no treatments for SOM approved by the U.S. Food and Drug Administration (FDA) for use in HNC or other cancers with solid tissue tumors. In the case of hematologic tumors, there is only one approved therapy (palifermin), a tissue growth factor which presumably encourages the growth and regrowth of the oral mucosa tissue. Palifermin is specifically approved for use in patients receiving hematopoietic stem cell support for a myelotoxic therapy of a hematologic cancer which lack the receptor for the growth factor [2], [3]. Due to its function as a tissue growth factor, palifermin is associated with a potential risk of stimulating/encouraging solid tumor proliferation [4], [5], [6] and is therefore contra-indicated in the case of solid tumors, all of which express the growth factor receptor. Other treatment approaches are under development [7], [8] but none have been approved and the risk of interfering with tumor treatment or encouraging tumor growth remains a primary concern [9].

Innate immunity is believed to play a key role in the pathogenesis of oral mucositis [10], [11], [12], [13] and indeed the efficacy of dusquetide as an Innate Defense Regulator supports this understanding [14], [15], [1]. Dusquetide (SGX942) is a first-in-class Innate Defense Regulator (IDR) that modulates the innate immune response downstream of most innate immune receptors, acting at a key adaptor protein known as p62 or sequestosome-1 [14]. Dusquetide modulates innate immune signaling from a pro-inflammatory, pro-macrophage response to an anti-inflammatory and increased pro-macrophage response. This response leads to decreased inflammation, increased bacterial clearance and increased tissue healing [14], [15], [16]. Importantly, dusquetide is not an anti-apoptotic or anti-necroptosis agent and cannot directly mitigate the damage done by CRT to the tumor [1].

Although direct interference with tumor therapy is unlikely, and indeed demonstrated not to occur preclinically [1], there are other potential ancillary effects of p62 interactions on tumor biology [17]. Specifically, p62 is a ubiquitous protein that is present in most cells, including aberrant tumor cells, and, through its role in autophagy, has been shown to impact tumorigenesis [18]. Thus, p62 is believed to be important in the tumorigenesis of MCF-7 (breast cancer cell line) where autophagy is otherwise inhibited [19], [20]. Again, a xenograft study with MCF-7 cells not only demonstrated a lack of interference with CRT but also demonstrated a lack of tumor enhancement with SGX942 treatment. In fact, reduction in tumor volume was observed preclinically with SGX942 treatment [1].

Innate immunity also plays a role in establishing the microenvironment around a tumor. For example, p62 has been directly implicated in facilitating the stromal cell microenvironment in multiple myeloma via a mechanism involving increased IL-6 signaling [21], [22].

To address the remote possibility that dusquetide may protect and/or enhance tumor growth, tumor resolution was monitored in the context of multiple myeloma cell growth in the presence of stromal cells and throughout a recent Phase 2 HNC study, both immediately after treatment and throughout a 12-month follow-up period. Similarly, overall survival of these HNC patients was also monitored through this same period.

## 2 Results and discussion

**Fig. 1:**
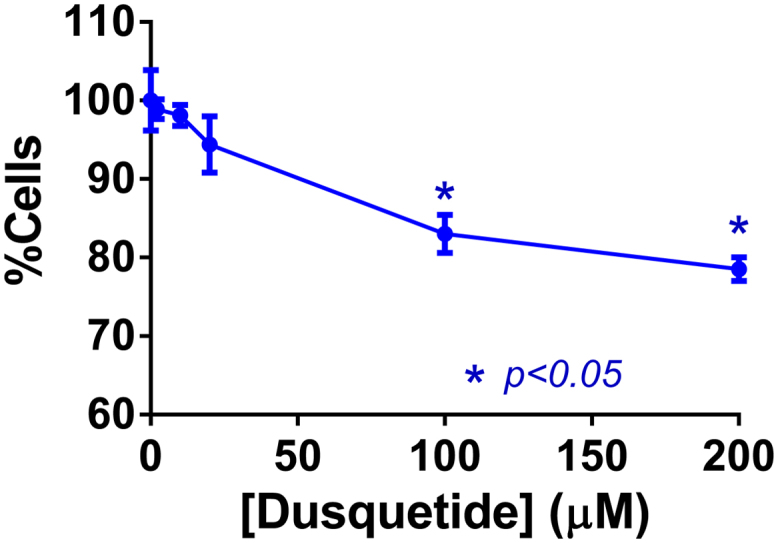
Dusquetide Pre-Incubated with Stromal Cells inhibits the Future Growth of Co-cultured Multiple Myeloma Cells. Previously defined primary human bone marrow stromal (mesenchymal (MSC)) cells (ReachBio lot# 2221207) were plated at a concentration of 10^4^ per well on 12 well plates in a McCoy’s based medium (Hyclone, lot# AVE72772) supplemented with 10% FBS (Hyclone, lot# ASF29773) and 2 mM L- Glutamine (Hyclone, lot# AUJ25591). These cells had been characterized by flow cytometry and the standard phenotype (CD45- CD34- CD73+ CD105+ CD90 + ) confirmed previously. The cells were used at passage 2 in all experiments. MM1.S cells (lot # 9000068) were purchased from ATCC and were cultured as recommended by the supplier in RPMI medium (Hyclone, lot# AVB62578), supplemented with 10% FBS (Hyclone, lot# ASF29773) and Glutamax (Gibco, lot# 889105) and allowed to expand. The marrow stromal cells were allowed to grow for 3 days. After this time, dusquetide was added at the indicated concentrations. Following 48 h incubation with dusquetide, the medium (and compound) was removed and the wells washed with RPMI containing 10% FBS and 2 mM L-Glutamine. To each well, 3 × 10^4^ MM1.S cells were added, and following an additional 48 h, these cells were removed by pipetting vigorously up and down and collecting the contents of each individual well into 5 mL tubes. Cell counts were performed without dilution using a Neubauer chamber.

Dusquetide was not expected to negatively impact the stromal microenvironment of multiple myeloma cells, since this signaling has been reported to rely on IL-6 signaling [21] and dusquetide has been shown to significantly reduce IL-6 [15]. Nonetheless, a co-culture system with human multiple myeloma cells was previously investigated and demonstrated that when dusquetide was pre-incubated with stromal cells, those same stromal cells provided reduced support for multiple myeloma cell growth (Fig. 1).

As reported previously [1], dusquetide (SGX942) reduced the median duration of SOM between initiation of CRT and the 1-month follow-up visit in a 111-patient double-blind placebo-controlled Phase 2 clinical trial of OM in HNC patients. In the patient population receiving at least 55 Gy irradiation, this reduction was 50% (18 days vs. 9 days in the placebo and SGX942 1.5 mg/kg treatment groups respectively, p = 0.099). In higher risk subpopulations, this reduction improved to 67% (30 days vs. 10 days in the placebo and SGX942 1.5 mg/kg group respectively [p = 0.040] in those patients receiving the highest doses of cisplatin chemotherapy). Ancillary measures during this initial window between initiation of CRT and the 1-month follow-up visit also demonstrated a decreased rate of non-fungal infection and an increased rate of “complete response” in the tumor assessments at the 1-month follow-up visit. The anti-infective and anti-inflammatory/tissue healing aspects of dusquetide were expected on the basis of previous preclinical work [15], [1].

**Fig. 2:**
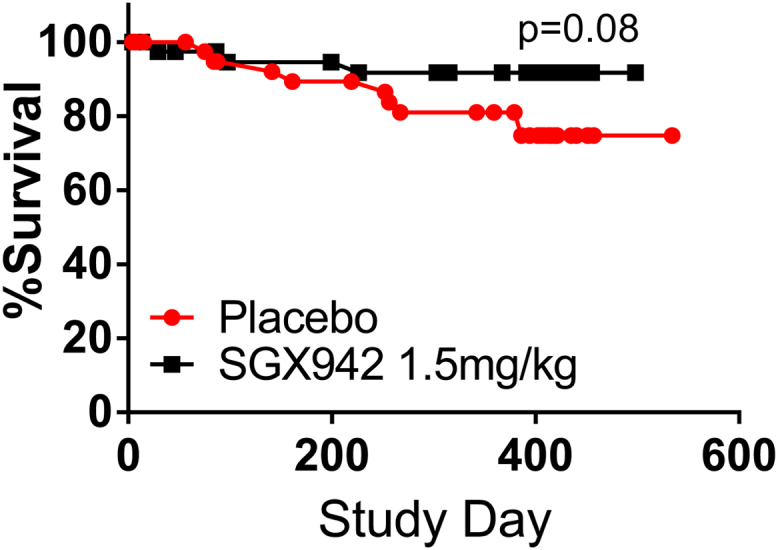
Kaplan-Meier Survival Curves from the Phase 2 IDR-OM-01 Study. The detailed study design is described in [1]. Survival was monitored for the 12 months following completion of chemoradiation therapy.

The one-year mortality rate in the placebo group was 81% (Fig. 2), consistent with the Surveillance, Epidemiology, and End Results statistics of approximately 80% survival for patients with tumors of the oral cavity, depending on tumor location [23]. In the dusquetide treatment groups, mortality was significantly lower. Using the pre-determined cutoff of p < 0.1 for statistical significance in this exploratory study yielded statistically significant improvement in survival using Kaplan-Meier analysis (Fig. 2), compared to placebo. This decreased mortality was primarily observed in the follow-up period (after the 1-month follow-up visit), suggesting it was not related to the decreased infection rate also observed in the dusquetide treated groups in this study prior to the 1-month follow up visit. While the underlying cause of the decreased mortality is unknown, these results certainly support the contention that SGX942 was safe and well-tolerated, without any identifiable negative side-effects in this patient population.

We previously reported a trend towards improved tumor resolution at 1-month post CRT using the RECIST v 1.1 criteria [24]. While it was not previously possible to ascertain if this improvement was transient or enduring, monitoring throughout the 12-month follow-up window demonstrated that the effect was in fact enduring (Table 1). Moreover, the patients in the placebo group continued to improve, eventually having a response rate more similar to the 1.5 mg/kg dusquetide treated group (Table 1). These results may indicate that dusquetide accelerated the tumor resolution. Given the size of this initial Phase 2 trial, it is impossible to ascertain if this improvement was due to increased compliance with CRT therapy or due to a direct effect of dusquetide on the tumor and its microenvironment. Given the known biology of p62 and the previously reported preclinical findings, it is possible that dusquetide had a direct anti-tumor effect in addition to reducing the duration of SOM.

**Table 1:** Tumor Progression as a Function of Elapsed Time since Completion of CRT^1^.

Timepoint	Placebo	1.5 mg/kg	3.0 mg/kg	6.0 mg/kg
SAFETY POPULATION (N)	41	42	3	23
1-month follow-up	15/32 (47%)	17/27 (63%)	1/3 (33%)	4/16 (25%)
LOCF^2^	26/35 (74%)	28/35 (80%)	1/3 (33%)	13/22 (59%)
mITT ^3^ POPULATION (N)	38	36	3	19
1-month follow-up	15/32 (47%)	17/27 (63%)	1/3 (33%)	4/16 (25%)
LOCF^2^	26/35 (74%)	28/34 (82%)	1/3 (33%)	12/19 (63%)

^1^Percentage calculation excludes missing/not assessed evaluations.

^2^Last Observation Carried Forward.

^3^modified Intent-to-Treat.

These results demonstrate that not only does dusquetide reduce the duration of a debilitating and burdensome side effect of most cancer treatment regimens, but that it could be associated with significant ancillary benefits including decreased infection rates and accelerated tumor resolution.

## Financial disclosure

The authors declare that they have competing financial interests in that two of the authors (RS, OD) are employees of Soligenix Inc., which is developing Innate Defense Regulators as human therapeutics.
